# Community-based workers’ capacity to develop inclusive livelihoods for youth with disabilities in Botswana

**DOI:** 10.4102/ajod.v10i0.851

**Published:** 2021-12-09

**Authors:** Ermien van Pletzen, Bryson Kabaso, Theresa Lorenzo

**Affiliations:** 1 Academic Development Programme, Centre for Higher Education Development, University of Cape Town, Cape Town, South Africa; 2 Division of Disability Studies, Department of Health and Rehabilitation Sciences, Faculty of Health Sciences, University of Cape Town, Cape Town, South Africa; 3 Department of Prosthetics and Orthotics, Princess Marina Referral Hospital, Gaberone, Botswana; 4 Division of Disability Studies, Department of Health and Rehabilitation Sciences, Faculty of Health Sciences, University of Cape Town, Cape Town, South Africa

**Keywords:** disability, youth, livelihoods, sustainable development, community-based rehabilitation, community development workers, environmental factors, Africa

## Abstract

**Background:**

Youth with disabilities encounter multiple barriers to livelihood opportunities and socio-economic inclusion. Research focusing on identifying and evaluating evidence-based strategies that may facilitate their transition into socio-economic participation is limited.

**Objectives:**

The study undertook to contribute knowledge and evidence to inform inclusive socio-economic development of youth with disabilities and capacitation of community-based workers engaged in implementing the livelihood component of community-based rehabilitation programmes advocating for inclusive development.

**Method:**

This qualitative exploratory case study used the International Classification of Functioning, Disability and Health: Children & Youth Version to analyse community-based workers’ knowledge and experience of the rural and peri-urban communities in which they worked in Botswana. It further analysed their activities, strategies and recommendations in response to environmental factors impacting the livelihood opportunities of youth with disabilities. Data were generated through semi-structured interviews, following a life history and phenomenological approach. Data were analysed inductively using thematic content analysis.

**Results:**

Community-based workers showed sufficient knowledge and experience of barriers and enablers in health, education and training, social development, employment and governance that facilitated or obstructed access to livelihood opportunities for youth with disability. Identifying more barriers than enablers, community-based workers adopted innovative strategies to sustain and strengthen their practices and activities in the livelihoods domain. They contributed recommendations, mainly aimed at government.

**Conclusion:**

Community-based workers have the capacity to provide valuable evidence and design strategy to facilitate the socio-economic inclusion of youth with disabilities. They are particularly adept at intervening at local levels but do not have sufficient confidence or capacity to mobilise supportive community structures or to exert influence at the level of policy formulation, decision-making and implementation.

## Introduction

Disability and poverty are intertwined in complex ways, flowing from and feeding into each other (Braithwaite & Mont 2009:230). Facilitating access to inclusive livelihoods is a powerful strategy to disrupt this cycle, which is especially effective if it enables youth with disabilities to establish a degree of socio-economic security early on in life (Chappell & Lorenzo 2012:20). However, research conducted in South Africa indicates that youth with disabilities are likely to experience multiple environmental barriers to accessing education and training, employment opportunities, support systems and leisure activities, all of which carry livelihood benefits (Lorenzo & Cramm 2012:581).

This situation also exists in Botswana despite its growing economy and relatively high per capita gross domestic product (Eide & Mattli 2016:16). Poverty rates remain high, whilst general income inequality (African Economic Outlook 2012:181) and high youth unemployment (Keetile 2014:340) persist. These conditions are far more severe for the 59 103 people with disabilities (2.9% of the overall population), particularly the 15 701 youth in the 15–34 year category (Mmatli, Kebotsamang & Lesetedi 2014:204–208). Researchers acknowledge the existence of policy and structures in Botswana that recognise the rights of persons with disabilities, such as the 1996 National Policy on Care for People with Disabilities and the Coordinating Office for People with Disabilities established in 2010 in the Office of the President (Mukhopadhyay & Moswela 2020:46–48; Omotoye 2018:1–9). However, researchers also point out that the country does not have disability-specific legislation entrenching equal opportunities in policies across all sectors of government. This makes it difficult to enforce the implementation of policy. The government has also not signed or ratified the United Nations Convention on the Rights of Persons with Disabilities (UN-CRPD 2006), which provides a framework for foregrounding the dignity and basic human rights of people with disabilities as equal members of society and for systematically mainstreaming disability issues as integral to strategies for sustainable development (Mukhopadhyay & Moswela 2020:46–47; Omotoye 2018:7; UN-CRPD 2006). Policy development has furthermore stalled in Botswana – revised disability policy drafted in 2011 to remove structural limitations to the meaningful integration of people with disabilities into social, economic, political and cultural aspects of life (Dinokopila & Mmatli 2014:22) has still not been accepted (Omotoye 2018:1). Within this context, research indicates that youth with disabilities continue to encounter multiple barriers to social inclusion and livelihood opportunities in Botswana, for instance, barriers to accessing education, vocational training and employment (Mmatli et al. 2014:204–208), whilst in the workplace attitudes of employers, peers and even family members hamper socio-economic inclusion (Mmatli 2007:283–284).

It has been shown that the adverse effect of disability on the well-being of youth can be mediated by employment and social support (Cramm, Lorenzo & Nieboer 2013:523). For this reason, providing evidence of methods and strategies including youth with disabilities in socio-economic development plans would be of significant social value. Such research would also strengthen the implementation and impact of community-based rehabilitation (CBR), the broad inclusive developmental strategy espoused by the World Health Organization that aims at providing equal opportunities to people with disabilities to access health services, education, livelihoods and social inclusion (WHO 2010a:11).

The central role played by community-based workers in the implementation of CBR has been widely acknowledged in research reporting on their practices, strategies and achievements. Researchers have shown the impact that they have on the lives of people with disabilities by creating support networks for the empowerment of individuals and their families in their communities (Chappell & Johannsmeier 2009). The strong alignment between their activities in diverse sectors and the WHO’s CBR guidelines has been highlighted (Deepak et al. 2011; Jansen-van Vuuren & Aldersey 2018). Their potential to alleviate poverty by increasing the levels of social, educational, economic and political inclusion of people with disabilities, their families and communities has been shown (Van Pletzen, Booyens & Lorenzo 2014), as well as their ability to harness their knowledge of complex rural contexts to improve the lives of people with disabilities (Booyens, Van Pletzen & Lorenzo 2015) and their competencies to make a contribution to social justice for persons with disabilities and their families by advocating on behalf of them (Lorenzo, Van Pletzen & Booyens 2015).

However, there is limited research focusing specifically on identifying and evaluating evidence-based methods and pathways that may facilitate the transition of youth with disability into socio-economic participation, which has a negative impact on designing appropriate developmental measures and strategies (Engelbrecht, Shaw & Van Niekerk 2017:6). Developing a clearer understanding of the conceptual complexity of disability and the diverse needs of youth with disabilities have been identified as a key requirement for improving public service providers’ capacity to design and implement targeted strategies that make inclusive development a reality, especially in resource-limited settings (Ned & Lorenzo 2016:6). Our study undertakes to help fill these gaps.

The WHO’s International Classification of Functioning, Disability and Health: Children & Youth Version (ICF-CY 2007) provides our study with a conceptual framework for interpreting community-based workers’ knowledge and experience, activities and practices, strategies and recommendations related to the socio-economic inclusion of youth with disabilities. The ICF-CY reinforces the Social Model of Disability, which underlies the shift in CBR programmes in the past 30 years. This involves a shift from primarily focusing on physical impairments and medical treatment to a focus on the social and environmental barriers that restrict a person’s functioning and the intersectoral strategies that could be adopted to equalise opportunities through rehabilitation, poverty reduction and social inclusion of people with disabilities (WHO & World Bank 2011:13).

Further conceptual substance was provided by the five components of the WHO’s CBR Guidelines (WHO 2010a:24–25), which detail the areas of inclusion for people with disabilities in health, education, livelihoods, social and empowerment domains. The livelihood component, the focus of this study, in turn comprises five elements: skills development, self-employment, wage-employment, financial services and social protection (WHO 2010b:7–8). With reference to the livelihoods component, Lorenzo, Motau and Chappell (2012:46) refer to community-based workers as ‘critical catalysts’ facilitating access to mainstream livelihood opportunities for youth with disabilities. The community-based workers who participated in this study were mid-level workers based in Botswana who have several years’ accredited training (Jansen-Van Vuuren & Aldersey 2018:7; Kabaso 2015).

The research question posed in this study is whether community-based workers have the capacity to provide evidence (to higher education institutions, government, researchers and service providers) that could contribute to improved measures and concrete strategies for facilitating inclusive livelihoods for youth with disabilities within mainstream sustainable development plans. Such evidence could also be used to capacitate community-based workers engaged in the livelihood component of CBR. The objectives of the study were to capture, analyse, interpret and evaluate community-based workers’ knowledge and experience of the rural and peri-urban communities they worked in; their activities, practices and strategies in response to the barriers and enablers that impact on the socio-economic inclusion of youth with disabilities and their recommendations to improve the socio-economic inclusion of youth with disabilities.

## Methodology

The study was nested in a larger research project that collected data from community-based workers (2011–2013) in Botswana, Malawi and South Africa (Booyens et al. 2015; Lorenzo et al. 2015; Van Pletzen et al. 2014). The nested study analysed data collected by a Botswana-based postgraduate student for a project which formed part of his Masters in Philosophy in Disability Studies from which a description of this methodology was drawn (Kabaso 2015). Like the larger study, the nested study had an exploratory case study design and adopted a qualitative, interpretive approach informed by life history research and phenomenology (Plummer 2001).

Data for the nested study were collected from three districts close to Gaberone in Botswana (Kabaso 2015). Districts were purposively selected for sharing characteristics of rural and peri-urban environments, also called ‘urban village’ environments by Eide and Mmatli (2016:8). Purposive sampling was used to select community-based workers from these districts. Initially, four participants were selected. Selection criteria were that participants should be conversant in English and have at least 5 years of experience working for government or non-governmental organisations (NGOs) addressing social and economic challenges facing people with disabilities in rural or ‘urban village’ environments. The same selection criteria were used at a later stage to select three further information-rich participants, with the added criterion that they should have specific experience addressing economic and livelihood challenges facing youth with disabilities in these environments. The overall sample of seven participants thus constituted two males and five females, one of whom had a disability.

Data were gathered through semi-structured individual interviews consisting of open-ended questions, with exploratory probes. The four participants selected initially were interviewed following a life-story approach focusing on participants’ experience of disability, career choice and key moments from their professional lives. Interviews focusing specifically on participants’ experience of facilitating livelihoods for youth with disabilities were subsequently conducted with two of the participants selected initially and the three participants selected at a later stage. These five participants all had rich experience of facilitating livelihoods for youth with disabilities.

All seven participants were informed of the purpose of the study, participation was voluntary, and they all gave informed consent. Interviews were conducted in English, and permission was requested to digitally record interviews. All interviews were transcribed verbatim. Confidentiality was protected by using pseudonyms to avoid revealing participants’ names or other identifiable aspects.

Interviews were analysed inductively using thematic content analysis. Themes related to community-based workers’ understanding and facilitation of the socio-economic inclusion of youth with disabilities were observed as they emerged, and further themes were identified until data saturation was reached. The data and themes were further interpreted using the ICF-CY (WHO 2007) and the five domains of the CBR livelihood component (WHO 2010b) as conceptual frameworks.

To verify the data, member checks were performed, either face to face or via e-mail and phone. This process provided the opportunity to ask for additional information and to ensure the credibility of the data. The authors conducted a joint participant and stakeholder workshop after data analysis was completed, allowing for further verification.

### Ethical considerations

Ethical approval was granted by the Health Research Unit of the Botswana Ministry of Health (Ref. No. PPME-13/18/1 Vol. VIII, 215) and by the University of Cape Town’s Faculty of Health Sciences Human Research Ethics Committee (Ref. No. HREC REF: 301/2013).

## Findings

**FIGURE 1:**
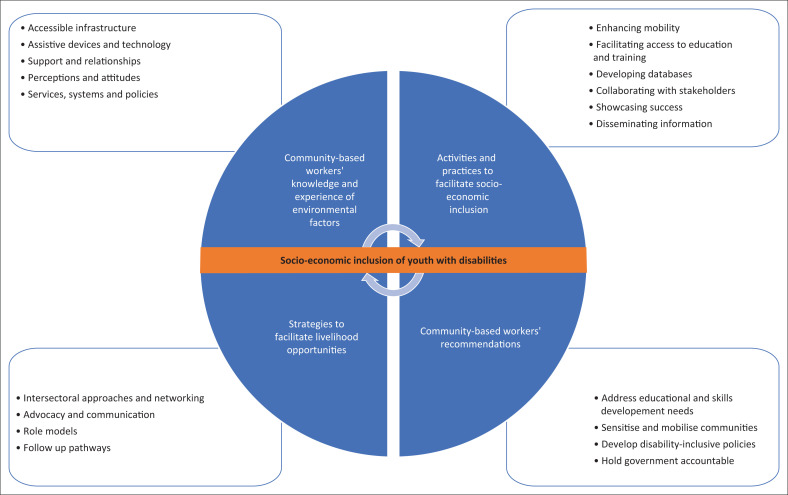
Key findings categorised in themes.

The findings are presented in four themes: community-based workers’ knowledge and experience of the rural and ‘urban village’ environments in which they worked, their activities and practices related to facilitating the socio-economic inclusion of youth with disabilities, their strategies and their recommendations. The key findings categorised under each of the four themes are presented in Figure 1.

### Community-based workers’ knowledge and experience of rural and ‘urban village’ environments

Participants showed substantial knowledge and experience of the enablers and barriers impacting on the socio-economic inclusion of youth with disabilities in rural and ‘urban village’ communities.

#### Overcoming physical barriers through assistive devices and technology

Participants showed awareness of the physical barriers that inaccessible infrastructure posed to youth with disabilities. Malebogo referred to a student who could not get to an upstairs venue and could therefore not ‘benefit like other students’ from the lecturer’s attention. Several participants, for this reason, stressed the importance of access to assistive devices and technology as a first step in overcoming physical barriers and facilitating socio-economic inclusion. Gil described a client who became a self-employed taxi driver after receiving a prosthesis, whilst Naledi illustrated how using rudimentary sign language became a bridge between her and youth who were deaf:

‘I haven’t received any training in sign language, but the little that I know, when I am with them, conversing with them, it’s nice because they will even joke or try to mimic what you are doing!’ (Naledi, Female, 32 years old)

#### They are ‘part of your life’: Support and relationships

Participants displayed an understanding of traditions in rural and ‘urban village’ communities that could lead to supportive relationships and closeness – ‘if there is someone with a disability… he or she is part of your life’ (Gil, Male, 36 years old). Traditions could however also become barriers to inclusivity: Kgomotso (Female, 45 years old) referred to stigmatising ‘beliefs that if you get in touch with them you will get children with disabilities’.

Lefika showed that excessively close family relationships could lead to over-protectiveness, which could become a barrier in itself:

‘You would find a disabled child is not involved in the household chores. Even if they are able to they are not being sent. Either it’s overprotection … but the basic thing is they become excluded from family activities.’ (Lefika, Male, 42 years old)

Naledi described the exclusion experienced by some of her deaf clients within their families:

‘There are also barriers with the parents, with the siblings….. They just want to be sitting there as a family chatting, not taking into consideration what this one is doing or thinking. They will not even do it in signs….’ (Naledi, Female, 32 years old)

Participants identified inadequate support for youth with disabilities also in the workplace. Lefika stated that ‘we end up losing people because they have not been properly supported within the system’. He further observed that:

‘Some of the companies also will definitely be underpaying, looking at the person and saying, “This is not going to be too much because I am doing this person a favour anyway,” and they pay little.’ (Lefika, Male, 42 years old)

#### ‘You know the culture’: Perceptions and attitudes

Participants illustrated how vague perceptions and blurred definitions of disability fed into negative attitudes, including stereotypes and stigma. Lefika expressed frustration with a blanket misunderstanding of disability:

‘People with disabilities are treated as people with special educational needs, special learning needs. So no matter [*if*] you are just in a wheelchair, looking to do academic things, they will feel you are a burden. (Lefika, Male, 42 years old)

He further pointed out stereotypes prevalent in the workplace:

‘Sometimes the reception work can be performed by someone with disabilities but … you know the culture, they want to put a nice looking lady at the reception and … where would they put these ones who maybe are in a wheelchair with deformity? They say the image of the organisation will be like that one….’ (Lefika, Male, 42 years old)

#### ‘Most of them are on paper really’: Inadequate services, systems and policies

Participants were particularly vocal in this category, mentioning enablers and barriers.

On the positive side, they clearly recognised the potential of a well-functioning inclusive education system to facilitate socio-economic inclusion of youth with disabilities. Gil told success stories of youth with disabilities who had attained high levels of education and found employment in social work and accountancy. Malebogo remarked that several technical colleges and universities had started admitting youth with disabilities and that some institutions provided disability services. She described a government system that broadened young people’s access by reducing standard admissions points.

On the whole, however, participants experienced many barriers to socio-economic inclusion in the public education and training system. At the broadest level, they found absence of information about disability a particularly pervasive problem: ‘People really … lack information, starting … from the councillors, schools … parents … they do lack information as far as disability ….is concerned’ (Gil, Male, 36 years old). They further highlighted the barrier posed by inaccessible and unavailable public transport services. Lefika (Male, 42 years old) found that successful placement of young people in educational institutions was undermined by the ‘problem of transport for people with disabilities. I have to struggle to take these people … to schools’.

Limited resources were frequently identified as a barrier. Malebogo (Female, 40 years old) observed that ‘even at primary level, most of these schools don’t have special education units’. Kgomotso (Female, 45 years old) explained that they had ‘only one unit’ for ‘over 80 children’ and no ‘qualified specialist teacher in this area’. The few post-primary institutions in existence were almost all run by NGOs, which hampered country-wide regulation of education and skills development. Centres were furthermore concentrated in urban districts. As a result, young people from rural areas were losing out on family contact (Malebogo, Female, 40 years old). Referring to children as young as three, Gil (Male, 36 years old) commented: ‘We are sort of killing … the social growth of a child. A child is now growing in an institute [*rather*] than growing with the family’.

The low levels and restrictive types of available education and training were mentioned. Kgomotso (Female, 45 years old) explained that youth with disabilities could often not ‘proceed to higher levels’ and experienced problems with ‘marketing themselves’. Naledi (Female, 32 years old) referred to the very limited skills development opportunities available at post-primary level to youth with intellectual disabilities: ‘they will just be sent to the community without anything’.

Available vocational training was found to be poorly aligned with the current socio-economic needs of the country: ‘the courses that they are offering are … almost the same for the whole country’ (Malebogo, Female, 40 years old). For this reason, participants struggled to find appropriate work placements for young people: ‘They have done textile in institutions … gardening, leather works. But now you see they are employed in a totally different area of operation’ (Kgomotso, Female, 45 years old).

When commenting on disability structures in Botswana, participants acknowledged some positives, but with many reservations. Masego expressed cautious appreciation of the ‘Office that is advocating for people with disabilities in the Office of the President’. Lefika (Male, 42 years old) praised the government’s adoption of ‘[*the*] Millennium Development Goals’ including ‘Universal Primary Education’, but pointed out that ‘the majority of our ex-students here … are not part of the 100%’. He also referred to ‘a policy on inclusive education’, which was, however, still at ‘policy level, implementation has not started’.

All participants were outspoken about the absence of clear policies and poor coordination of disability structures and services. Malebogo pointed to a problem at the highest political level:

‘as a country we have not signed the UN Convention [*on the Rights of People with Disabilities*] …. I think … if the country can sign that, then a lot of things will be in place … but for now everything is offered from different corners.’ (Malebogo, Female, 40 years old)

Naledi blamed confused government structures for weaknesses in service delivery. She ascribed social workers’ reluctance to engage with disability issues to general structural confusion:

‘At our district, the programme was with DHMT first [*the District Health Management Team*], then it was transferred, I think it’s 3 years back, to Social and Community Development. So they still have that problem with assisting the youths with disabilities.’ (Naledi, Female, 32 years old)

Gil (Male, 36 years old) referred to the government’s instruction to ‘all departments to have a disability committee’, adding: ‘the thing is the committees are not functional, they are not doing the work that they are supposed to do’.

Participants, for instance, Kefilwe, were vocal about the failure of policies to translate into practice:

‘There are policies and there are structures, they are there, but it’s just written in black and white…. There isn’t any law enforcement…. You will have a policy saying that this ministry should do this or these particular people should do this, but even if they don’t do it there wouldn’t be any legal … actions taken against them.’ (Kefilwe, Female, 31 years old)

Even though participants critiqued the failure of policy, they were also prepared to admit that they did not have high levels of policy literacy and were often ignorant about current policy. Kgomotso acknowledged community-based workers’ responsibility to translate policy into practice:

‘Most of them are on paper really…. we still need to go out there in the kgotla [*traditional community council*] meetings and … talk to people about these policies because … people are not very much aware of or sensitised about the policies that we are talking about.’ (Kgomotso, Female, 45 years old)

Gil (Male, 36 years old) confessed, ‘I have realised that policies are there but … [*they*] are in the shelves’.

Community-based workers’ extensive knowledge and experience of rural and ‘urban village’ communities informed and shaped their activities and practices, as will be shown in the second theme.

### Activities and practices to facilitate the socio-economic inclusion of youth with disabilities

#### Enhancing mobility and facilitating physical access

Participants saw enhancing mobility and facilitating physical access to health and educational services as important pre-conditions for socio-economic inclusion. Naledi advocated for ramps at government facilities to ensure access to general social and community development services. Gil described collaborating with social workers, who provided lifts for children needing to undergo assessment for educational placement.

#### Facilitating access to education and training

Participants further described multiple activities and practices related to facilitating access to education and training. Kgomotso outlined steps in multi-layered referral and placement processes: identifying youth with potential, assessing the nature and level of their disability and investigating appropriate educational opportunities. They would assist the few who qualified for tertiary education by helping prospective students with application and admission procedures. For those who had not ‘gone that far’, they would gather information and assist with placement procedures at appropriate Brigades (colleges providing technical and in-service training) or rehabilitation centres offering basic training. For youth with more severe disabilities, usually involving intellectual disabilities, they would ‘liaise with our colleagues at Social and Community Development’ for places on short courses in areas such as ‘textile, crocheting … bead making and so forth’.

#### Developing databases of youth with disabilities for placement purposes

Naledi mentioned a successful practice of matching a list of available opportunities with a database of people with disabilities for speeding up verification:

‘If … there is a disabled person maybe who is applying for an empowerment scheme or a project, maybe at the Youth Department, they will want clarification from my office that … in fact this is a person with disability.’ (Naledi, Female, 32 years old)

#### Collaborating with partners and stakeholders

Several participants described the collaboration with a government initiative encouraging the private sector to employ people with disabilities. Malebogo (Female, 40 years old) explained: ‘They will ask us, “Who are the people with disabilities around … that we can employ?” Then we send their CVs … and they screen them’.

Kgomotso described moving between government and prospective employers to secure wage employment for promising candidates:

‘So what we have done, we have applied with them [*the youth*], they have submitted their names to Office of the President, and Office of the President has a liaising Officer or Human Resources officer at [*Name of Supermarket*] headquarters. So after submitting, what we do is we keep phoning them: “Have you not secured a place somewhere where you can put our clients who have applied with your office?” So that’s the little that we have managed so far.’ (Kgomotso, Female, 45 years old)

#### Showcasing success

Participants realised that showcasing successful employees with disabilities could counteract stereotypes. Lefika mentioned that some employers had ‘tested’ the potential of youth with disabilities and found that they were ‘more productive than the … able bodied ones’. He recalled placing some youth with disabilities at a local industry, where ‘despite … challenges’ they were found to be ‘more honest and it was better productivity than before’. One of them was even *‘*chosen to be the workers’ steward … because … they saw the passion, they saw the interest’. Lefika felt that ‘contrary to people’s beliefs, [*this youth*] proved them wrong’. After such experience, employers would ‘actually request: “We want people with disabilities to come and work in our establishment”’.

Counteracting stereotypes could open up new types of opportunities. Lefika mentioned that ‘when these diamond polishing companies were being established, they would say “We want so many people with visual, with hearing impairment, to be part of our team”’. He recalled a young person missing upper limbs who was employed at a company as an administrator: he could ‘write faster than most of these able bodied’. When he moved to the United States of America to study for a degree, the company ‘insisted on looking for someone with disabilities’, saying ‘We have never seen productivitiy of this magnitude’.

#### Disseminating information about training and livelihood opportunities

Despite limited social protection measures in Botswana, participants were active in disseminating information and connecting disabled youth with the few available opportunities. Gil arranged for the National Youth Council to ‘educate the people with disabilities about the programmes that they give so that at least they could benefit from the government’. He further described using donor grants to finance the start-up of micro-businesses for five young people with visual impairment who had only primary level education. They were provided with chickens, basic equipment and feed. Apart from gaining in ‘confidence’, ‘esteem’ and ‘morale’, ‘some are even saying they are willing to go back to school’.

The practices and activities presented in this section are sustained by systemic strategies that community-based workers devised, as can be seen in the third theme.

### Strategies to facilitate livelihood opportunities for youth with disabilities

Five systemic strategies were mentioned by participants to facilitate the socio-economic inclusion of youth with disabilities.

#### Finding ‘people that can help us’: Networking and intersectoral approaches

Participants described intersectoral approaches as key strategies in facilitating livelihood opportunities for youth with disabilities. They described themselves engaging in internal and external networking, liaising across multiple sectors and levels of practice.

Gil described how an intersectoral approach routinely shapes his daily activities: working directly with disabled youth and their families, assessing their ‘economic potential’ and ‘situation’, educating them about available government programmes, helping them to ‘come up with income generation projects’, liaising with government departments to organise workshops, calling on the National Youth Council to provide education on available opportunities and at a more practical level, assisting young people ‘to write business proposals’ and then to e-mail proposals ‘to people that can help us’ (various stakeholders such as donors, employers, government institutions or NGOs).

#### ‘You should know which office or which door to knock [on]’: Advocacy and communication

Participants’ accounts of their practice showed the importance of advocacy as a strategy, as for instance in Gil’s case: ‘As a community-based worker, you should be able to advocate… you should know which office or which door to knock [*on*]*…*’. He further mentioned using inventive, multi-layered approaches to strengthen advocacy and disseminate information, for instance, by working through *kgotlas* (traditional councils) and community chiefs to present educational workshops and information sessions on disability issues. He harnessed the power of the media, appearing on Botswana Television, which ‘was really an eye opener’ for the public, resulting in a flood of phone calls and visits from people needing information about education and livelihood opportunities for disabled youth. He also successfully promoted education and training centres for disabled children by using Radio Botswana to cover a wide geographic area. This resulted in 75 children joining their rehabilitation centre.

#### Creating hope: Using role models to empower youth and promote employability

Role modelling was highlighted as a strategy to empower youth with disabilities and to promote their employability. Lefika explained how community-based workers used role modelling to ‘create hope’: when youth with disabilities see the success of peers, they start thinking: ‘Well, we have something to look forward to’. Successful peers were a source of inspiration: ‘To some extent it has created a platform for people to be brave, daring’. Role models could also convince employers to give more youth with disabilities employment opportunities: ‘Some have really opened their eyes to say, “Well, this is the right thing to do”’.

#### Creating follow-up pathways

Malebogo described a careful process of integrating young employees with disabilities into the work environment by getting employers to agree to ‘try’ one or two candidates, who are then supported through follow-up visits:

‘Then you coach those two time and again, and also visit them time and again, to see whether they are doing the right thing, and also try to talk to the manager time and again until they are stable at work.’ (Malebogo, Female, 40 years old)

In this way, community-based workers kept in touch with former clients and projects whilst creating the opportunity of steering the development of youth with disabilities, as well as giving advice and support to them and their employers.

The final theme draws together community-based workers’ expertise in a set of recommendations, which are based on the evidence presented by the previous themes.

### Community-based workers’ recommendations for enhancing disability-inclusive livelihood opportunities

Participants made a number of recommendations that they felt would support the socio-economic participation of disabled youth.

#### Address the educational and skills development needs of youth with disabilities

The scarcity of specialised government-run training institutions was seen as a major obstacle in securing livelihoods for disabled youth. One of the recommendations was for more government institutions. Participants also recommended the development of a cohort of specialised, qualified teachers. Malebogo stated: ‘We … need personnel, people who are trained to work with people with disabilities, and we need centres’. Gil emphasised the importance of providing decentralised training. There was also a recommendation for the government to provide more relevant forms of vocational skills training for youth with disabilities, and Malebogo recommended that young people should be consulted on their choice of training: ‘Look at their interests … you just take them and say, “Go and do leather work!” After that they are not interested in leather work!’

#### Sensitise and mobilise communities

The importance of sensitising communities as a way of empowering youth with disabilities was observed. Malebogo spoke of the need to ‘build their self-esteem’ and showed how this required community awareness of environmental factors:

‘We … need the whole community, for everybody, to be aware of the problems that people with disabilities have, the barriers that make them not to achieve like other able-bodied person.’ (Malebogo, Female, 40 years old)

Participants recommended putting youth with disabilities and their communities at the centre of developmental planning. In the first place, ‘You will learn a lot when you go into the community’, for instance, that ‘people with disabilities can even teach you, they are [*the*] best teachers, they will tell you their experiences’. Lefika argued that:

‘You need to find out what it is the community there really needs, and plan with the community…. That is very important. If you don’t plan with the community … and [*merely*] take yours [*plans*] there, it becomes your programme and they won’t support it much…. You need community involvement.’ (Lefika, Male, 42 years old)

#### Develop up-to-date disability-inclusive policy, appropriate definitions and coherent structures

Participants emphasised the need for up-to-date disability-inclusive policy. Whilst Malebogo appreciated that there was a new disability policy in draft form, she pointed out that the country’s 1996 disability policy was outdated. She further asked for clearer bench marking and definition:

‘We are registering people with disabilities and issuing them with a card, [*but*] there is no standard…. When we say a person is disabled, what is disability and what are the types, categories of disability?’ (Malebogo, Female, 40 years old)

She mentioned that as community-based workers they had ‘advised on bench-marking’, but the process was allocated to Sweden, instead of a country that more resembled conditions in Botswana, like South Africa.

Several participants highlighted the need for more coherent government structures. Naledi expressed frustration with fragmentation: ‘At the Health Ministry there is something about disability, at Local Government ministry there is something about disability’. Malebogo observed: ‘We need to be in one ministry and speak with one voice and know … the services that we are offering to people with disabilities’.

#### Make government accountable for developing livelihood opportunities for youth with disabilities

Several participants were in favour of government endorsing a quota system for employment of youth with disabilities. Kgomotso proposed ‘a clause’ that would bind ‘both Government and Non-Governmental Organisations to … employ maybe 5% of people with disabilities’. Naledi recommended ‘that affirmative action be fully utilised’, because ‘disability is hampering the livelihood’ of this group. For youth with severe disability, Kgomotso recommended the introduction of sheltered employment. Salaries would never be as high as in the open market, ‘but it’s still better, you know, they will still get something’.

In summary, the findings present multifaceted evidence about community-based workers’ knowledge and experience, their activities and practices, as well as the strategies that they devised to remove environmental barriers and enhance environmental enablers. They further suggested useful recommendations to facilitate inclusive socio-economic development of youth with disabilities.

## Discussion

The data show that community-based workers in this study had sufficient knowledge and experience of the rural and ‘urban village’ communities that they worked in to be able to identify key environmental factors that enabled and obstructed disabled youth’s access to livelihood opportunities. Identifying more barriers than enablers, community-based workers adopted innovative strategies to sustain and strengthen their work in the livelihoods domain. Their recommendations were mainly aimed at government although they acknowledged that they themselves needed to acquaint themselves with relevant policy developments and challenges of implementation.

**FIGURE 2:**
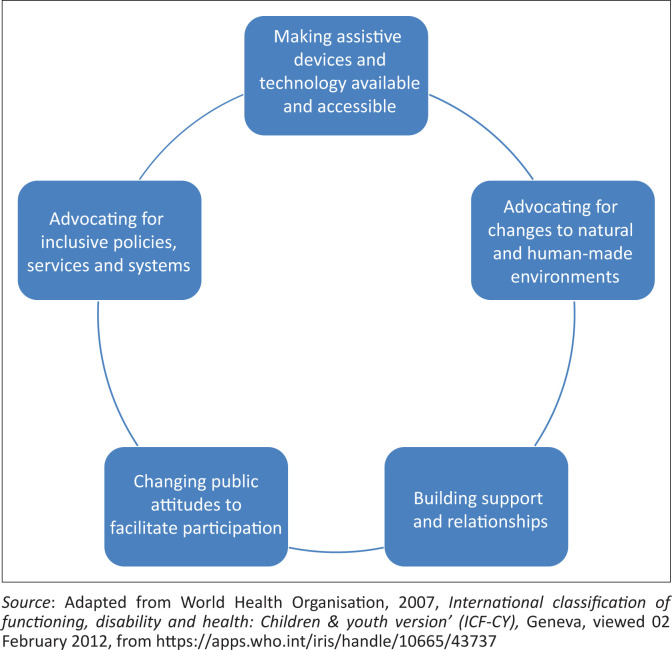
Measures and strategies linked to the International Classification of Functioning, Disability and Health: Children & Youth Version categories of environmental factors.

The ICF-CY’s five categories of environmental factors (WHO 2007:28) provide a framework for returning to the research question whether community-based workers have the capacity to provide evidence that could contribute to improved measures and concrete strategies for facilitating inclusive livelihoods for youth with disabilities within mainstream sustainable development plans. These factors include the ‘physical, social and attitudinal environment in which people live and conduct their lives’, which could either obstruct or facilitate individuals’ functioning (WHO 2007:xviii; 9). Figure 2 provides a visual summary of measures and strategies linked to the ICF-CY’s categories of environmental factors that emerged from the evidence.

### Making assistive devices and technology available and accessible

Community-based workers realised the potential of assistive devices to improve the chances of youth with disabilities to become socio-economically active. Community-based workers played an important role in identifying young people who would benefit from assistive devices, provided information and networked with donors and professionals to improve access. In this respect, they were aided by the Botswana government’s relatively good performance in providing assistive devices mainly through the public health service and mostly free of charge (Matter & Eide 2018:3–4).

However, research also shows that the need for assistive devices in Botswana remains largely unmet (Matter & Eide 2018:7) and that, as in many low- to middle-income countries (LMICs), the availability of mobility devices (such as wheelchairs, crutches and prostheses) predominates, with much less access for people with impairments in hearing, seeing, communication or cognition (Eide & Mmatli 2016; Matter & Eide 2018). The literature also points to the need for holistic national plans regulating equitable access to assistive devices and technology (Borg, Lindström & Larsson 2011:26). The study data reflect this situation. Only one community-based worker spoke of the inclusive value of sign language and regretted not having received any training in it. Other participants focused exclusively on facilitating mobility. This finding indicates that community-based workers’ knowledge and practice relating to providing equitable access to a range of assistive devices and technology urgently need to be expanded and regulated.

### Advocating for making the natural and human-made environment accessible

Community-based workers showed awareness of the roles played by the natural and human-made environment in either facilitating or obstructing inclusion in educational and employment opportunities for youth with disabilities. In Botswana, as in many other resource-restricted countries, the design of educational buildings does not accommodate disability (Mukhopadhyay et al. 2012:6), whilst the built environment also becomes a major obstacle to inclusion in the workplace (Mmatli 2007:280). There was evidence in the study of participants advocating for more accessible environments on the ground. However, community-based workers in the study did not seem to have the confidence or competency to interact with planners or political decision-makers at higher levels. This barrier echoes a finding by Chappell and Johannsmeier (2009:9–10; 12) that community-based workers’ impact was mainly at the private, individual level. In this respect, it becomes evident that community-based workers’ low levels of policy literacy and ignorance about current policy have the effect of hampering their ability and confidence to enforce inclusive policy in the public and political spheres.

### Building support and relationships

Community-based workers were able to broker supportive relationships in families, communities and the workplace by offering assistance, information and counselling and by advocating for youth with disabilities. Their first-hand knowledge and experience of rural and ‘urban village’ communities equipped them well for this task. They focused not only on creating multilayered support networks but also on counteracting stigmatising or other obstructive attitudes. Their experiences and practices are in line with research, which indicates that whilst families are a fundamental source of strength and support, they can also be limiting and obstructive when family members undermine the confidence of youth with disabilities or doubt their effectiveness in the workplace (Singal & Jain 2012:171). Employers’ attitudes could further be discouraging because they often expect and accept poor performance from employees with disabilities and do not support their progression (Mmatli 2007:284).

### Changing public attitudes to facilitate participation

Community-based workers in the study can be seen working across multiple sectors – health, education, transport, the private and public labour market – to counteract negative attitudes that create barriers to socio-economic inclusion. Their strategy of show-casing the strengths and abilities (rather than the difficulties) of youth with disabilities was particularly successful in this respect, opening up new forms of employment and demand in the open labour market and contributing towards dismantling employment discrimination and negative attitudes from employers, supervisors and peers (Stuart 2006). Other studies likewise show the impact of beliefs about the causes of disability and how they could erect barriers to participation for people with disabilities (Maart et al. 2007:366). Studies also show how community-based workers could counteract such barriers, for instance, by recognising that taxi drivers’ stigmatising beliefs could be significant barriers to accessible public transport, which in turn could influence livelihood opportunities (Lorenzo & Cramm 2012:580).

### Advocating for inclusive policies, services and systems

Community-based workers showed awareness of the challenges experienced by youth with disabilities across various sectors of service and government. They grappled with the limitations of policy, insufficient mainstreaming of disability issues and difficulties encountered in the coordination of disability services.

Participants acknowledged education, at all levels, as an important factor impacting the socio-economic participation of youth with disabilities. Children and youth with disabilities have worse access to education and attain far lower levels of education than the non-disabled, whilst the quality of education that they receive is also almost always inferior (Dinokopila & Mmatli 2014:1). In combating these challenges, participants tended to advocate for more schools and training centres specifically targeting young people with disabilities, rather than for more widespread availability of inclusive education. Research shows that inclusive education remains available to only small numbers of learners with disabilities, and that there are serious limitations to implementing inclusive models, especially in resource-restricted settings (Mukhopadhyay, Johnson Nenty & Okechukwu 2012:6–9; Zwane & Malale 2018:8–11). Community-based workers could benefit from developing a better understanding of the potential gains of inclusive education (Kuper, Saran & White 2018:15–16). They could play a role in strengthening inclusive education by raising awareness of different educational options and assisting youth with disabilities to navigate between options (Howgego, Miles & Myers 2014:12).

Responding to the challenge of under-employment or unemployment amongst youth with disabilities, community-based workers liaised with representatives from the private sector and the Office for People with Disabilities to open up new employment opportunities on the open job market. An area where community-based workers in the study seemed to have less capacity was in strengthening and collaborating with Disabled People’s Organisations (DPOs) to empower youth with disabilities and strengthen their ability to advocate for themselves (Mmatli 2007:288).

Whilst community-based workers were vocal in expressing their frustrations with policy and systemic weaknesses, their knowledge of policy issues and the importance of mainstreaming disability across multiple sectors tended to be vague and mainly theoretical, whilst their capacity for action at policy level was generally weak. One participant (Naledi) expressed frustration with fragmentation of disability services across multiple ministries. However, another participant’s (Malebogo’s) desire for locating all disability services in ‘one ministry’ would undermine integration and inclusion, which are key concepts in rights based and equal opportunities approaches to disability that emphasise the importance of mainstreaming disability issues as integral to strategies for sustainable development (UN 2006).

More consistent and legally enforced implementation of a human rights-based approach and stronger coordination of disability services across the sectors would better resolve the policy and structural difficulties experienced by community-based workers than the centralisation of specialised disability services. Whilst Botswana is not a signatory of the CRPD (UN 2006), the country’s disability policy recognises and protects the disability rights and dignity of individuals (Mukhopadhyay & Moswela 2020:47). However, few of the country’s policies make specific provision for disability or define disability clearly. There is also no specific legislation offering comprehensive legal protection for people with disabilities (Dinokopila & Mmatli 2014:33), a situation that puts people with disabilities at the mercy of service providers (Mukhopadhyay & Moswela 2020:46). Translation of policy into practice has moreover been slow (Omotoye 2018:10–11), suggesting that several of the factors characterising ‘policy evaporation’ (Alfred & Harrison 2006:6) could be at play in Botswana: insufficient political commitment, complexities presented by mainstreaming, inadequate funding and insufficient guidance during implementation. An important step in implementation has been disability coordinating committees established at district level by the Office for People with Disabilities to improve intersectoral collaboration amongst stakeholders and develop partnerships with businesses to facilitate employment opportunities for people with disabilities (Omotoye 2018:11). However, the coordinating Office and district committees lack authority and capacity, again pointing to an urgent need for legislation that would empower the Office to enforce its mandate of monitoring whether government departments and other stakeholders are playing their respective roles in ensuring the socio-economic inclusion of people with disabilities (Omotoye 2018:50). Whilst these represent challenges that community-based workers commented on, or even opportunities that they exploited, they seemed to lack confidence and skill to exert pressure on policymakers and implementers. Research in neighbouring countries points to similar challenges experienced at the level of policy formation, implementation, enforcement and service coordination (Chichaya, Joubert & McColl 2018; Dziva, Shoko & Zvobgo 2018). Equipping community-based workers with a more nuanced understanding of complex policy and systemic issues, as well as the skills necessary to engage in these environments, could strengthen their advocacy role in mainstreaming disability and their capacity to facilitate more systemic access to livelihood opportunities for youth with disabilities.

## Recommendations

Our study prompts a number of recommendations. Firstly, community-based workers’ knowledge of the range of assistive devices and technology available, their understanding of how these could facilitate the functioning of youth with disabilities and their ability to assess and refer children and youth to specialised services need to be expanded beyond the current focus on supporting mobility. Training should be adjusted to include more information on assistive devices and technology supporting impairments in hearing, seeing, communication, self-care and cognition. Community-based workers should also be trained to identify and respond to the large unmet need for assistive devices and technology across different types of impairments.

Secondly, community-based workers’ understanding of the barriers posed by the natural and human-made environment and possible interventions need to be expanded similarly to extend beyond a focus on mobility restriction.

Thirdly, community-based workers should receive more training in sensitising and mobilising the public and systematically expanding and collaborating with support and empowerment networks for youth with disabilities. Such networks should include DPOs and should make provision for special interest groups focusing particularly on the socio-economic participation of youth with disabilities. This empowerment would strengthen capacity to advocate for more disability-inclusive resources, policy, legislation and implementation, at both local and national levels.

Finally, community-based workers need to become more informed about existing policies, systems, services and regulatory mechanisms, which need to be disability inclusive, with the intention of assessing and identifying gaps and then prioritising responses. They should also be capacitated to develop an understanding of the governance structures related to disability services and support systems, particularly those related to the ability to grasp the types of governance structures and requirements for coordination that facilitates disability inclusion in national strategies and programmes. Accessible disability-inclusive research and policy translation toolkits should be designed by teams of community-based workers, members of DPOs and disability researchers, drawing on local and international research. These toolkits should be disseminated widely through print and digital means.

## Conclusion

Our analysis of the viewpoints and experiences of a cadre of community-based workers active in rural and ‘urban village’ environments in Botswana demonstrates their capacity to influence the socio-economic inclusion of youth with disabilities. The study reveals that through training and practice community-based workers in resource-constrained environments can develop the capacity to act as ‘catalysts’ (Lorenzo et al. 2012) in facilitating the socioeconomic inclusion of disabled youth. The findings further suggest that community-based workers are particularly adept at intervening at local levels, which Max-Neef (1991) argues is where people-centred development will achieve poverty reduction and social inclusion.

A number of challenges emerged from the study. Whilst participants show that they have some knowledge of the policy environment and sufficient understanding of implementation challenges to make pertinent recommendations on how to improve livelihood opportunities for youth with disabilities, the study reveals that community-based workers themselves do not currently have confidence or capacity to mobilise supportive community structures (such as DPOs or faith-based organisations) or to exert influence at the level of policy formulation, decision-making or implementation. This is an area that requires further investigation and needs to be addressed in the basic and further professional training of community-based workers.
